# Association of β-Amyloid Accumulation With Executive Function in Adults With Unimpaired Cognition

**DOI:** 10.1212/WNL.0000000000013299

**Published:** 2022-04-12

**Authors:** Pontus Tideman, Erik Stomrud, Antoine Leuzy, Niklas Mattsson-Carlgren, Sebastian Palmqvist, Oskar Hansson, for the Alzheimer's Disease Neuroimaging Initiative

**Affiliations:** From the Clinical Memory Research Unit, Department of Clinical Sciences (P.T., E.S., A.L., N.M.-C., S.P., O.H.), and Wallenberg Center for Molecular Medicine (N.M.-C.), Lund University; and Memory Clinic (P.T., E.S., S.P., O.H.) and Department of Neurology (N.M.-C.), Skåne University Hospital, Sweden.

## Abstract

**Background and Objectives:**

The neuropathologic changes underlying Alzheimer disease (AD) start before overt cognitive symptoms arise, but it is not well-known how they relate to the first subtle cognitive changes. The objective for this study was to examine the independent associations of the AD hallmarks β-amyloid (Aβ), tau, and neurodegeneration with different cognitive domains in cognitively unimpaired (CU) individuals.

**Methods:**

In this cross-sectional study, CU participants from the prospective BioFINDER-2 study were included. All had CSF biomarkers (Aβ42 and phosphorylated tau [p-tau]181), MRI (cortical thickness of AD-susceptible regions), Aβ-PET (neocortical uptake), tau-PET (entorhinal uptake), and cognitive test data for memory, executive function, verbal function, and visuospatial function. Multivariable linear regression models were performed using either CSF Aβ42, p-tau181, and cortical thickness or Aβ-PET, tau-PET, and cortical thickness as predictors of cognitive function. The results were validated in an independent cohort (Alzheimer’s Disease Neuroimaging Initiative [ADNI]).

**Results:**

A total of 316 CU participants were included from the BioFINDER-2 study. Abnormal Aβ status was independently associated with the executive measure, regardless of modality (CSF Aβ42, β = 0.128, *p* = 0.024; Aβ-PET, β = 0.124, *p* = 0.049), while tau was independently associated with memory (CSF p-tau181, β = 0.132, *p* = 0.018; tau-PET, β = 0.189, *p* = 0.002). Cortical thickness was independently associated with the executive measure and verbal fluency in both models (*p* = 0.005–0.018). To examine the relationships in the earliest stage of preclinical AD, only participants with normal biomarkers of tau and neurodegeneration were included (n = 217 CSF-based; n = 246 PET-based). Again, Aβ status was associated with executive function (CSF Aβ42, β = 0.189, *p* = 0.005; Aβ-PET, β = 0.146, *p* = 0.023), but not with other cognitive domains. The results were overall replicated in the ADNI cohort (n = 361).

**Discussion:**

These findings suggest that Aβ is independently associated with worse performance on an executive measure but not with memory performance, which instead is associated with tau pathology. This may have implications for early preclinical AD screening and outcome measures in AD trials targeting Aβ pathology.

The shift from a symptom-based to a biological definition of Alzheimer disease (AD), within the research framework,^1^ has highlighted the importance of biomarkers indicative of the neuropathologic changes underlying AD. A deeper understanding of how neuropathologic changes relate to cognition could potentially have implications for early identification of AD and for outcome measures in clinical trials targeting these changes. Depositions of β-amyloid (Aβ) is considered one of the characteristics of AD and the accumulation starts decades before the clinical syndrome appear.^2-4^ The association between Aβ and cognition in cognitively unimpaired (CU) individuals has been elusive in cross-sectional studies,^5-8^ whereas longitudinal studies more robustly have shown an Aβ-related cognitive decline over time.^9-11^ Another hallmark of AD is the presence of aggregated tau, which shows a connection with memory loss.^12-14^ Tau is also associated with atrophy, which in turn is related to deficits in neuropsychological test performance.^15,16^ Previous studies have often focused on how 1 or 2 of the pathologic processes are associated with cognition in the preclinical stage. This could have obscured independent associations. We therefore investigated how the distinctive pathologies of AD and neurodegeneration are independently related to cognition in CU. Both CSF- and PET-based biomarkers were used. As a secondary aim, we examined the relationship between Aβ pathology and cognition in individuals with no signs of abnormal tau or atrophy present to identify the first cognitive deficits associated with pathologic change in AD. Analyses were first performed in the BioFINDER-2 study and then validated in an independent cohort (Alzheimer’s Disease Neuroimaging Initiative [ADNI]).

## Methods

### Participants

For this study, we included CU participants from the ongoing prospective Swedish BioFINDER-2 study (NCT03174938), described in detail elsewhere.^17^ The study enrolls participants in the Southern Healthcare region of Sweden (Region Skåne, at Skåne University Hospital, and the Hospital of Ängelholm) and includes cognitively healthy controls; patients without dementia with cognitive symptoms subsequently categorized as subjective cognitive decline (SCD), mild cognitive impairment (MCI), or AD with dementia; and patients with other neurodegenerative diseases. The present study only included CU participants. In accordance with the National Institute on Aging–Alzheimer’s Association (NIA-AA) classification,^1^ participants with SCD were grouped together with cognitively healthy controls. To be considered SCD (and not MCI), participants had to perform within normal limits (a deviation of less than −1.5 SD) according to age- and education-stratified norms in all measured cognitive domains (supplementary eMethods 1, links.lww.com/WNL/B743). The inclusion and exclusion criteria are given in eMethods 2. Individuals who had a complete baseline dataset of CSF Aβ42, CSF phosphorylated tau (p-tau)181, MRI, Aβ-PET, tau-PET, and neuropsychological assessment were included. One presymptomatic microtubule-associated protein tau (MAPT) mutation carrier was excluded, resulting in a total of 316 CU participants. Data for this study were collected between April 2017 and October 2019.

### Standard Protocol Approvals, Registrations, and Patients Consents

All participants gave written informed consent and ethical approval was given by the Regional Ethical Committee in Lund, Sweden. Approval for PET imaging was obtained from the Swedish Medical Products Agency.

### AT(N) System

The NIA-AA has proposed a classification system where the underlying neuropathologic processes of AD, rather than the symptoms, are defining the disease. The AT(N) system divides biomarkers into 3 categories based on the pathologic process they measure: Aβ (A), tau (T), and neurodegeneration (N). All individuals who are positive for A are considered to be within the AD spectrum; those with concomitant abnormal T (A+T+[N]− or A+T+[N]+) are considered to have AD.^1^

### CSF Analyses

CSF Aβ42 and CSF p-tau181 was quantified using Innotest immunoassay (Fujirebio). Aβ positivity was defined using a cutoff of <783.7 pg/mL for CSF Aβ42 and tau positivity using a cutoff of >58.8 pg/mL for CSF p-tau181. Both cutoffs were derived using mixture modeling^18^ based on CSF levels from all participants who had undergone lumbar puncture at baseline in the BioFINDER-2 cohort by October 2019 (n = 949).

### MRI Procedures

Structural MRI was performed using a Siemens 3T MAGNETOM Prisma Scanner (Siemens Medical Solutions). T1 images underwent volumetric parcellation and segmentation using FreeSurfer (v. 6.0). An AD-signature cortical thickness meta–region of interest (ROI), recommended for FreeSurfer thickness extraction method, was used as a biomarker for AD-like neurodegeneration.^19^ The meta-ROI encompasses temporal and parietal regions with known susceptibility to atrophy in AD (mean thickness in the bilateral entorhinal cortex, inferior temporal, midtemporal, inferior parietal, fusiform, and precuneus regions). The cortical thickness measure was binarized using a cutoff (<2.50 mm) derived from mixture modeling^18^ based on all available data by October 2019 from participants in the BioFINDER-2 cohort who underwent MRI at baseline (n = 415).

### Aβ- and tau-PET

Aβ-PET imaging was acquired on digital GE Discovery MI scanners (GE Healthcare) 90–110 minutes after the injection of [^18^F]flutemetamol. The standardized uptake value ratio (SUVR) was calculated with pons as reference region. Tau-PET imaging was performed on the same PET scanners 70–90 minutes post injection of [^18^F]RO948, as previously described in detail.^20^ SUVR were created using the inferior cerebellar cortex as reference region.^21^ In order to capture brain regions affected early by Aβ and tau pathology, volume-weighted FreeSurfer-based ROI composites were created. The early-stage Aβ-PET composite included the precuneus, medial orbitofrontal, and posterior cingulate cortices, where early accumulation of Aβ occurs.^22,23^ For the early-stage tau-PET ROI, a composite including transentorhinal and entorhinal regions, corresponding to Braak stages I and II, was used.^24^ To establish cutoffs for Aβ and tau positivity, Aβ-PET and tau-PET data were binarized using cutoffs (Aβ-PET >0.558 SUVR and tau-PET >1.368 SUVR) derived from mixture modeling^18^ based on all participants in the BioFINDER-2 study cohort with Aβ-PET and tau-PET data at baseline (n = 438 and n = 975, respectively) available by October 2019.

### Neuropsychological Assessment

All participants completed a neuropsychological test battery (eMethods 1, links.lww.com/WNL/B743). Four measures were selected to maximize agreement between test batteries in the BioFINDER-2 and ADNI cohorts, each one representing a cognitive function from the different domains known to be affected in AD.^25^ The 10-word delayed word list recall from the Alzheimer's Disease Assessment Scale (ADAS) was used as a measure of episodic memory. Animal fluency was used as a measure of verbal fluency and cube analysis from the Visual and Object Perception Battery (VOSP) was used for visuospatial ability. The Trail-Making Test B (TMT-B) minus the Trail-Making Test A (TMT-A) difference score (TMT B-A) was used to measure an aspect of executive functions. Subtracting the time to complete the TMT-A from the time to complete TMT-B renders a relatively pure measure of the more complex divided attention and altering sequencing tasks of part B while reducing visuoperceptual and working memory demands, thus providing an indicator of executive control abilities, as previously described.^26,27^ For all tests, raw scores were used, except for the TMT B-A score, which was skewed and therefore log transformed. To avoid negative differences, a value of 10 was added to all TMT B-A scores prior to log transformation. ADAS delayed recall score indicates number of missed words, animal fluency score indicates number of animals within 1 minute, and VOSP cube analysis score indicates number of correct tasks. Larger TMT B-A score implies worse executive performance. The correlations between the measures used were small to medium, with ADAS delayed recall and animal fluency having the highest correlation (*r* = −0.363) (eTable 1).

### Validation Cohort: ADNI

Data used in the preparation of this article were obtained from the ADNI database (eMethods 3, links.lww.com/WNL/B743). All cognitively normal participants with a complete baseline set of neuropsychological assessment, CSF, and MRI data that had passed quality control in the temporal and parietal regions, *APOE* coded as presence/absence of an ε4 allele, white matter lesions volume (WML), diabetes, and hypertension status were included, resulting in a population of 361 participants (eMethods 4). The specific inclusion/exclusion criteria for the ADNI cohort can be found online in the ADNI database. ADNI was approved by the institutional review boards of all participating institutions and written consent was obtained from all participants at each site.

CSF levels of Aβ42 and p-tau181 were measured using the Elecsys assay (Roche Diagnostics GmbH). Both CSF biomarkers were binarized using previously published cutoffs (Aβ42 <1,100 pg/mL and p-tau181 >28.69 pg/mL).^28,29^

For MRI, T1-weighted images underwent volumetric parcellation and segmentation using FreeSurfer and the same AD-signature cortical thickness meta-ROI as described above was used. The cortical thickness measure was binarized using a cutoff (<2.60 mm) derived from mixture modeling^18^ using all participants with a screening visit MRI (n = 1,342; data files UCSFFSX51_ADNI1_3T_02_01_16.csv, UCSFFSX51_08_27_19.csv, and UCSFFSX6_02_05_20.csv).

In the ADNI cohort, the same cognitive tests as in BioFINDER-2 were used except for VOSP cube analysis, which is not a part of the ADNI assessment battery and therefore was replaced by clock copying. The TMT B-A score had a skewed distribution and was log transformed; raw scores were used for the other tests. All test scores were retrieved from the NEUROBAT.csv file or the ADNIMERGE.csv file.

### Binarization of Biomarkers

In the analyses, Aβ was binarized (normal/abnormal) based on previous findings showing that the levels of CSF Aβ42 (or the Aβ42/Aβ40 ratio) drop relatively sharply when amyloid starts accumulating in the brain but does not continue to decrease as the accumulation continues^30^ (i.e., it measures the gradual Aβ accumulation poorly and acts more like a binary marker of Aβ status). To establish cutoffs, gaussian mixture modeling was used, which is a robust way of determining thresholds in an unbiased way (i.e., the diagnosis or status is not used in analysis) and has been extensively applied previously.^18,31-33^ To examine the effect of Aβ on cognition in the earliest stage of the AD continuum, biomarker profiles were established by binarizing tau and cortical thickness using mixture modeling statistics (in order to select a sample that were T− and N− for the secondary analysis). Density plots for the different biomarkers are provided in eFigure 1 (links.lww.com/WNL/B743). For the ADNI sample, previously published cutoffs for Elecsys CSF Aβ42 and p-tau181 were used, while the cortical thickness measure was established using mixture modeling, as no previous cutoffs were available.^28,29^

### AT(N) Biomarker Profiles

To examine the relationship between Aβ pathology and cognition in individuals with no signs of abnormal tau or atrophy, all participants were categorized according to the AT(N) system^1^ (A−T−[N]−, A+T−[N]−, A+T+[N]−, A+T+[N]+, A+T−[N]+, A−T+[N]−, A−T−[N]+, A−T+[N]+) dependent on their biomarker profile using CSF Aβ42 as A, CSF p-tau181 as T, and AD cortical thickness meta-ROI as N. In a secondary analysis, the participants from BioFINDER-2 were instead categorized using Aβ-PET as A, tau-PET as T, and the AD-signature cortical thickness meta-ROI as N.

### Statistical Analyses

Multivariable linear regression models were used to examine the independent effects of Aβ, tau, and neurodegeneration on cognition. The different cognitive measures were used as dependent variables in the separate models; amyloid (CSF Aβ42 or Aβ-PET), tau (CSF p-tau181 or tau-PET), and cortical thickness (AD-signature meta-ROI) were used as independent variables. CSF Aβ42 and Aβ-PET were treated as binary variables; CSF p-tau181, tau-PET, and cortical thickness were used as continuous variables in the models. Age, sex, and education were also included as covariates.

In the first step, analyses were performed on participants with all possible combinations of AT(N) biomarkers, first using CSF Aβ42, CSF p-tau181, and cortical thickness (CSF-based model). Then, Aβ-PET, tau-PET, and cortical thickness (PET-based model) were used as biomarkers for AT(N), to test if the results were robust across biomarker modalities. To specifically examine the effect of Aβ on cognition in the earliest stage of the AD continuum, participants with either no positive biomarker (A−T−[N]−) or with just positive Aβ (A+T−[N]−) were included. To control for possible effects of tau and cortical thickness, these variables were also included, as well as age, sex, and education. Multivariable linear regression models were first performed in the BioFINDER-2 study and then in the ADNI cohort, using the same variables and covariates. The rationale for carrying out the same analyses in ADNI was to validate the results in an independent cohort with different characteristics and hence to minimize the risk of false-positive results. A *p* value of <0.05 was considered significant. Standardized β with 95% CI are reported. All analyses were performed in SPSS v 25 (IBM). The package “mixtools”^18^ in R version 4.0 was used for mixture modeling analysis (R Foundation for Statistical Computing). Voxelwise implementation of multilinear models was performed using SPM version 12 software.

### Data Availability

Anonymized study data for the primary analyses presented in this report are available upon reasonable request from any qualified investigator for purposes of replicating the results.

## Results

A total of 316 CU participants were included from the Swedish BioFINDER-2 study. Table 1 presents the demographics and characteristics of the BioFINDER-2 population. Out of the 316 participants, 54.4% and 68% were classified as A−T−(N)− when using either the CSF-based model (i.e., CSF Aβ42, CSF p-tau181, and cortical thickness) or the PET-based model (i.e., Aβ-PET, tau-PET, and cortical thickness), respectively. The A+T−(N)− subgroup consisted of 14.2% CSF and 9.8% PET (Table 2).

**Table 1:**
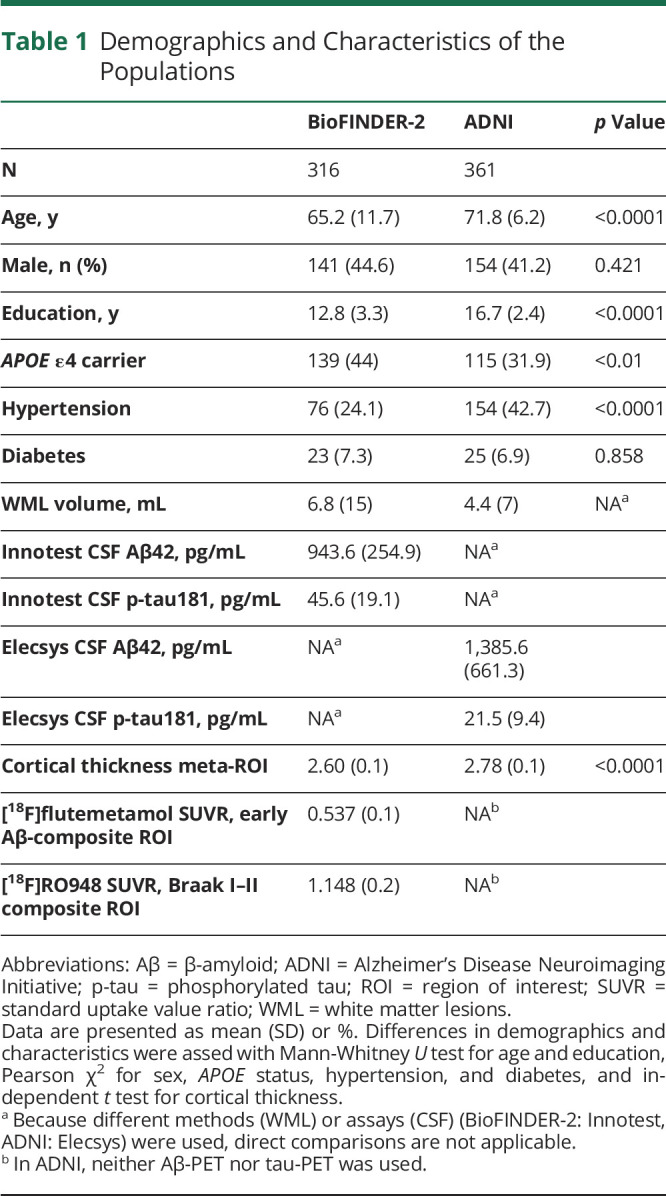
Demographics and Characteristics of the Populations

**Table 2:**
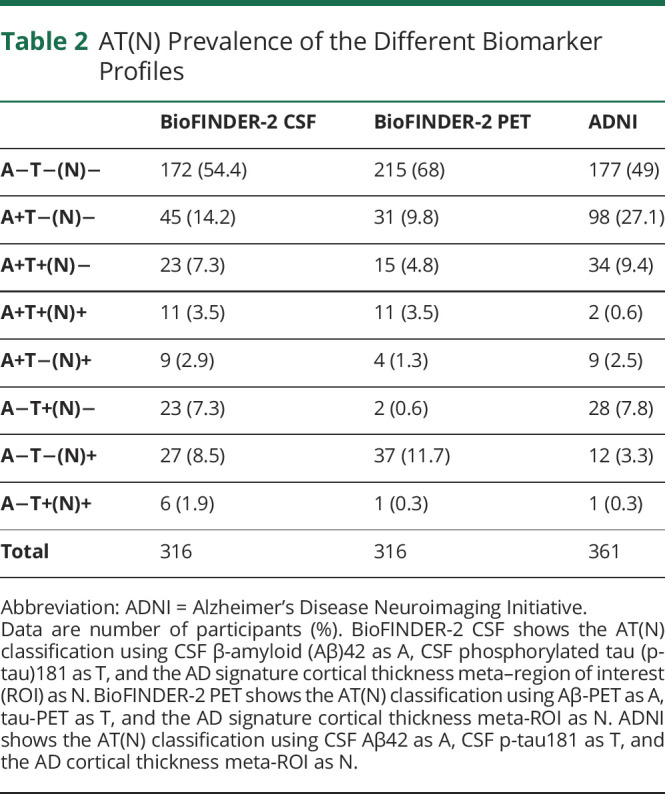
AT(N) Prevalence of the Different Biomarker Profiles

### Independent Associations of Aβ, Tau, and Cortical Thickness With Cognition in the BioFINDER-2 Cohort

**Figure 1:**
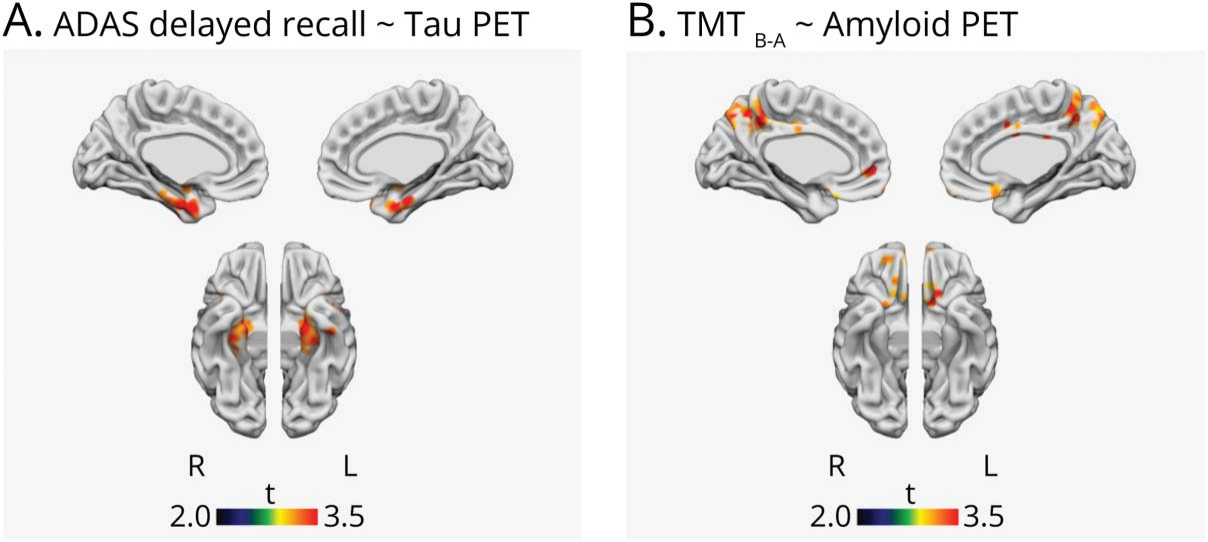
Voxelwise Multilinear Models Results from voxelwise multilinear models showing (A) the association between memory (Alzheimer's Disease Assessment Scale [ADAS] delayed recall) and tau-PET standard uptake value ratio (SUVR), adjusted for β-amyloid status, cortical thickness, age, sex, and education; and (B) the association between executive function (Trail-Making Test B–A difference [TMT B-A]) and amyloid-PET SUVR, adjusted for tau, cortical thickness, age, sex, and education. Statistical maps were thresholded at *p* < 0.005, k > 50.

Independent effects of Aβ, tau, and cortical thickness on cognition were tested first in CSF-based model and then in the PET-based model. The results are shown in Table 3. In the total BioFINDER-2 sample, there was an association between worse performance on the executive measure (TMT B-A difference) and abnormal Aβ status using CSF Aβ42 (β = 0.128, *p* = 0.024), adjusted for CSF p-tau181 and cortical thickness as well as for age, sex, and education (demographics). The association between abnormal Aβ status and worse performance on the executive measure was also found using Aβ-PET (β = 0.124, *p* = 0.049), adjusted for tau-PET SUVR, cortical thickness, and demographics. Memory (ADAS delayed recall) was not associated with Aβ status in either model (CSF *p* = 0.219; PET *p* = 0.775). Memory was instead associated with tau measured either using CSF p-tau181 levels (β = 0.132, *p* = 0.018) or tau-PET SUVR (β = 0.189; *p* = 0.002), adjusted for Aβ status, cortical thickness, and demographics. Voxelwise analyses confirmed the ROI-based findings with tau-PET, showing positive associations between memory and [^18^F]RO948 SUVR in the medial temporal lobe (*p* < 0.005, k > 50) (Figure 1A). CSF p-tau181 levels, but not tau-PET SUVR, were also associated with verbal fluency (animal fluency) (β = −0.123, *p* = 0.037). Cortical thickness was independently associated with the executive measure (β = −0.161, *p* = 0.013; β = −0.186, *p* = 0.005) and verbal fluency (β = 0.163, *p* = 0.011; β = 0.156, *p* = 0.018), both in CSF-based and PET-based models, adjusted for Aβ status, tau, and demographics. None of the biomarkers showed any independent associations with visuospatial performance (VOSP cube analysis) in these CU participants.

**Table 3:**
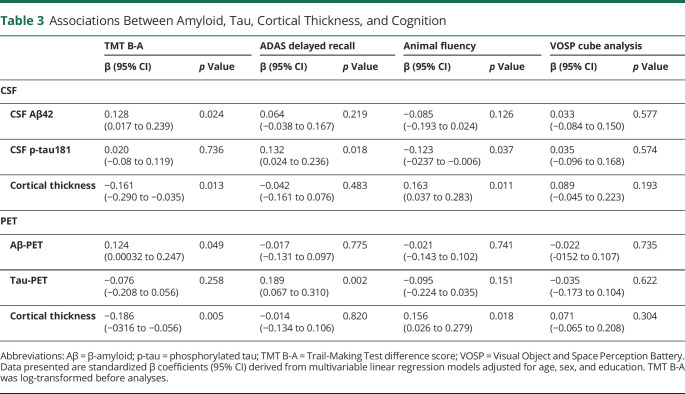
Associations Between Amyloid, Tau, Cortical Thickness, and Cognition

In the second step of analyses, aimed at identifying the first cognitive deficits associated with a pathologic change in AD, we specifically examined the relationship between Aβ pathology and cognition in individuals where no signs of abnormal tau or neurodegeneration were present (i.e., using the A−T−[N]− and A+T−[N]− subgroups; n = 217 for CSF-based model, n = 246 for PET-based model). Again, the only significant association between abnormal Aβ status and cognition was seen for the executive function measure (TMT B-A difference), either using CSF Aβ42 (β = 0.189, 95% CI 0.057–0.321; *p* = 0.005) or Aβ-PET (β = 0.146, 95% CI 0.021–0.273; *p* = 0.023), adjusted for tau, cortical thickness, and demographics. Voxelwise implementation of the PET-based model revealed similar findings in the form of positive associations between TMT B-A scores and [^18^F]flutemetamol SUVR in the posterior cingulate/precuneus and orbitofrontal cortex (*p* < 0.005, k > 50) (Figure 1B). None of the other cognitive measures showed any associations with any of the biomarkers (eTable 2, links.lww.com/WNL/B743).

### Validation of the Results in the ADNI Cohort

CU participants (n = 361) from ADNI (Table 1) were used to validate results from the CSF-based model. Multivariable linear regressions with the same type of variables as in the BioFINDER-2 cohort (i.e., CSF Aβ42 status, p-tau181 levels, cortical thickness, and demographics) also showed an independent association between abnormal Aβ status and worse performance on the executive measure (TMT B-A difference; β = 0.148, 95% CI 0.048–0.247; *p* = 0.004). Furthermore, tau was marginally associated with memory (β = 0.099, 95% CI −0.003 to 0.200; *p* = 0.056). Finally, cortical thickness was independently associated with executive function (β = −0.165, 95% CI −0.273 to −0.057; *p* = 0.003). None of the biomarkers showed any associations with any of the other cognitive measures (eTable 3, links.lww.com/WNL/B743).

When ADNI participants with no signs of abnormal tau or atrophy were included in the analyses (i.e., the A−T−[N]− and A+T−[N]− subgroups; n = 275), an association was again found only between abnormal Aβ status and worse performance on the executive measure (β = 0.143, 95% CI 0.023–0.262; *p* = 0.019); increasing CSF p-tau181 levels were associated with worse memory performance (β = 0.180, 95% CI 0.066–0.293; *p* = 0.002). None of the other cognitive measures showed any associations with any of the biomarkers (eTable 4, links.lww.com/WNL/B743).

### Sensitivity Analyses

When applying correction for multiple comparisons (false discovery rate [FDR] at the 0.05 level), the associations of CSF Aβ42 status and TMT B-A, cortical thickness and TMT B-A (both models), and tau-PET and ADAS delayed recall were still significant in the total BioFINDER-2 sample, and the association between Aβ42 status and TMT B-A was also significant after FDR correction in the subgroup analysis (the A−T−N− and A+T−N− sample). Because cardiovascular risk factors (hypertension and diabetes) and WML (eFigure 2, links.lww.com/WNL/B743) have been associated with cognition,^34^ these were also adjusted for in another sensitivity analysis along with *APOE* genotype, to examine whether this would influence the associations. Results were overall similar to what was previously found (eTables 5–8). That is, Aβ was still significantly associated with the executive measure both in BioFINDER-2 and ADNI. To examine whether an association between Aβ and the executive measure was observed when Aβ was used as a continuous variable, we also used continuous CSF Aβ42 instead of a binary CSF Aβ42 variable. The results were similar, showing a significant association between continuous CSF Aβ42 and the executive measure (total BioFINDER-2 sample: β = −0.115, 95% CI −0.224 to −0.006, partial *r* = −0.118, *p* = 0.038; A−T−(N)− and A+T−(N)− sample: β = −0.202, 95% CI −0.472 to −0.067, partial *r* = −0.20, *p* = 0.003; see eTable 9).

## Discussion

This study examined how the different hallmarks of AD (Aβ, tau, and neurodegeneration) independently are associated with cognition in CU adults in 2 independent and deeply phenotyped cohorts. In the Swedish BioFINDER-2 study, we found that Aβ pathology, measured using either CSF or PET, was independently associated with performance on the executive measure, but not memory, verbal fluency, or visuospatial function. This effect of Aβ pathology on the executive measure was also found when only examining participants without biomarker evidence of tau pathology or neurodegeneration. Furthermore, both CSF- and PET-based measures of tau pathology were independently associated with memory performance. Finally, neurodegeneration, measured using MRI, was associated with performance on the executive measure and verbal fluency. The results from the BioFINDER-2 study were overall replicated in the independent ADNI cohort. Taken together, these results suggest that, on the AD continuum, early Aβ accumulation is associated with decreased performance on an aspect of executive function while tau is associated with memory performance.

The relationship between Aβ accumulation and executive function found in this study was seen independently of tau and cortical thickness and regardless of which Aβ biomarker that was used. Previous studies in CU populations have reported small but significant associations of Aβ and specific cognitive domains, foremost highlighting an association between Aβ and memory but also a relationship between increased Aβ burden and executive function.^6,35,36^ The current results are thus partly consistent with previous findings.

Executive function is considered an umbrella term, which encompasses a set of different abilities. In this study, we utilized a delta score (TMT B-A) that reduces visuoperceptual and working memory demands for the task. Based on the measure used in this study, the results suggest that neuropsychological tests tapping more complex divided attention and cognitive flexibility are perhaps more sensitive to Aβ accumulation. TMT, and especially TMT-B, is widely considered a test of frontal lobe function. But as with other tests within the executive domain, the task is multifaceted, and executive performance is also dependent on other regions of the brain.^37,38^

The underlying cause of the effect of Aβ on specifically executive function is unknown, but one explanation could be a regional direct effect of Aβ accumulation. The early accumulation of Aβ has been shown to affect connectivity within different functional networks, primarily the default mode network but also the central executive network (CEN), which comprises the dorsolateral prefrontal cortex and posterior parietal cortex.^23,39^ CEN is engaged in attention and mental flexibility^40,41^ and it is possible that the altered connectivity within the CEN due to Aβ is causing the executive deficit exhibited by the TMT B-A difference. Instead of a regional effect, it could also be caused by a disruption of cholinergic neurotransmission, which has been suggested to occur as a result of Aβ accumulation.^42^ The cholinergic system is involved in attention and is especially important when increased attentional effort is needed.^43,44^ As aforementioned, the TMT-B is an attentional challenging task and the association between the TMT B-A difference score and Aβ positivity found in this study could possibly reflect a cholinergic deficiency due to the effects of Aβ on the cholinergic neurotransmission. Another possibility is that when Aβ burden has reached a threshold, it is associated with a subtle effect on different cognitive functions. This discrete effect might be captured by the TMT B-A difference because it is probably the most sensitive of the measures used and if more challenging tasks tapping other domains had been administered, they, too, could perhaps have captured this effect. Partial correlations were found between continuous Aβ42 and performance on the executive measure, both in the total sample and in the A−T−(N)− and A+T−(N)− subgroup (*r* = −0.118, *p* = 0.038; *r* = −0.20; *p* = 0.003), which is similar to what has previously been reported.^35^ The association of Aβ on executive function found in this study seems to be of a small, but significant, magnitude, which implies that other factors influence the variability in the TMT B-A measure, perhaps explained by intellectual differences (e.g., fluid cognitive ability), intrapersonal factors (e.g., alertness, day-to-day differences in performance), or possibly by other pathologies (e.g., TDP-43), not accounted for in the models.

As for the effect of early tau accumulation (increased CSF p-tau181; increased tau-PET signal in transentorhinal and entorhinal cortices) on cognition, we found that it was associated with memory, which is in line with previous findings.^12,13^ Tau pathology, especially in the transentorhinal and entorhinal cortices, seems to be related to impaired episodic memory^45^ and these are the regions where neurofibrillary tangles initially appear in AD.^46^ There is a strong association between tau and neurodegeneration and both are related to impaired cognition.^47^ In this study, however, we found an independent association between tau and memory. This could indicate that subtle memory impairment is more related to tau than atrophy in CU individuals, which is in line with the development of biomarker abnormalities during the AD trajectory, where tau precedes atrophy.^48^

This study did not confirm the relationship between Aβ and memory shown in previous studies.^6,8,35^ There could be different explanations for this disparity. First, previous studies did not study the independent effects of Aβ on memory function by adjusting for tau pathology. Considering that Aβ and tau are associated in AD, it is likely that Aβ acts as a surrogate marker for tau in many study participants with early AD, when tau is not accounted for in the models. Another possibility is that Aβ is related to decreased attentional control, which subsequently may influence the encoding phase of the memory process, and use of the delayed recall measure in this study might not have captured this effect.

Neurodegeneration, measured with the cortical thickness composite ROI encompassing temporal and parietal regions, was independently associated with performance on the executive function measure in the total BioFINDER-2 sample and in the total ADNI sample. The temporo-parietal composite used contains regions especially susceptible to atrophy in AD and these regions partially overlap with the brain regions that exhibit changes in activation when performing the TMT task.^49^ The independent effects of both Aβ and atrophy on executive function are congruent with the hypothesis that early Aβ pathology, which affects the function of synapses,^50^ has an early negative effect on certain aspects of executive function, which is subsequently exaggerated by loss of neurons (as reflected by brain atrophy on MRI). This is in contrast to memory function, which was only related to medial temporal lobe tau in this CU population.

A strength of this study was that we examined the contribution of Aβ, tau, and AD-like cortical atrophy simultaneously in multivariable models. The results found were similar in the 2 cohorts despite differences in CSF assays, age, education, and how participants were recruited. Nevertheless, this study has several limitations. The PET-based results were not validated in the ADNI cohort because of few participants with all variables. In ADNI, only 60 CU participants (by February 2020) had a complete baseline dataset of CSF, MRI, Aβ-PET, tau-PET, and neuropsychological assessment; especially tau-PET was the limiting biomarker. Another limitation is that the study design does not allow for a causal interference of Aβ accumulation and worse executive function since only cross-sectional data were used. Furthermore, the observed association of Aβ and executive function was rather modest, which may limit its clinical utility. This said, future studies should further explore if existing tests or novel tests that measure a similar cognitive function as the TMT B-A score could be more sensitive to early Aβ pathology. Such tests might potentially be used for screening of preclinical Alzheimer pathologic change in CU individuals, especially if used longitudinally at an intraindividual level. Because it seems that the TMT B-A score detects an effect on cognition that is independently related to Aβ accumulation, a more sensitive executive measure could perhaps also be useful to include in clinical trials targeting Aβ, as performance of memory tests appears to primarily reflect the effect of tau on cognition.

Aβ-PET and tau-PET composites were defined a priori, as well as the composite for cortical thickness. The selection of composites was based on previous findings indicating regions that are early and typically affected in the AD continuum.^19,23^ However, as this approach allows for a reduced number of comparisons, it may also limit the possibility to discover how different spatial depositions or atrophy patterns are related to different cognitive functions. Especially the AD-signature composite encompassing several regions might have obscured a more regional, specific effect of neurodegeneration on cognitive function.

We found evidence of an independent relationship between early Aβ accumulation and worse performance on the executive measure in CU individuals. Tau was independently associated with memory performance whereas the AD-signature cortical thickness measure was related to the executive measure. Although the reported associations between Aβ and the TMT B-A difference score are modest, this finding could contribute to the understanding of how cognitive deficits are manifested in asymptomatic individuals with early Aβ accumulation and more sensitive measures of this cognitive ability might be useful for screening for Aβ pathology or used as an outcome measure in clinical trials targeting Aβ.

## Glossary

Aβ: β-amyloid
AD: Alzheimer disease
ADAS: Alzheimer's Disease Assessment Scale
ADNI: Alzheimer’s Disease Neuroimaging Initiative
CEN: central executive network
CU: cognitively unimpaired
FDR: false discovery rate
MCI: mild cognitive impairment
NIA-AA: National Institute on Aging–Alzheimer’s Association
p-tau: phosphorylated tau
ROI: region of interest
SCD: subjective cognitive decline
SUVR: standard uptake value ratio
TMT-A: Trail-Making Test A
TMT-B: Trail-Making Test B
TMT B-A: Trail-Making Test difference score
VOSP: Visual Object and Space Perception Battery
WML: white matter lesions
